# Serum Trace Elements in Patients With Polycystic Ovary Syndrome: A Systematic Review and Meta-Analysis

**DOI:** 10.3389/fendo.2020.572384

**Published:** 2020-09-17

**Authors:** Jiechen Yin, Xiang Hong, Jun Ma, Yuanqing Bu, Ran Liu

**Affiliations:** ^1^Key Laboratory of Environmental Medicine Engineering, Ministry of Education, School of Public Health, Southeast University, Nanjing, China; ^2^Key Laboratory of Pesticide Environmental Assessment and Pollution Control, Nanjing Institute of Environmental Science, Ministry of Ecology and Environment, Nanjing, China

**Keywords:** PCOS, trace elements, meta-analysis, zinc (Zn), copper (Cu), magnesium (Mg), iron (Fe)

## Abstract

Polycystic ovary syndrome (PCOS) is reported to be associated with certain trace elements. However, previous data are inconsistent and potentially biased due to small sample sizes. The potential utility of trace element levels for screening of PCOS remains to be established. The aim of this meta-analysis was to investigate the potential relationships between PCOS and serum levels of zinc (Zn), copper (Cu), magnesium (Mg), iron (Fe) and ferritin. We carried out a literature search of PubMed, EMBASE, and Web of Science for relevant cross-sectional/case-control studies published prior to October 2019. Random-effect models were used to estimate the overall standard mean differences (SMDs) between PCOS and healthy control subjects. The screening value of potential microelement biomarkers for PCOS was assessed using the receiver operating characteristic (ROC) curve. Twenty-one studies featuring 2,173 women with PCOS and 1,897 healthy women were selected for analysis. Our results showed that Cu and ferritin levels were significantly higher in women with PCOS than healthy controls, with SMDs of 0.52 [95% confidence interval (CI): 0.38–0.67, *I*^2^ = 47.6%] and 1.05 (95% CI: 0.25–1.86, *I*^2^ = 97.0%), respectively. The serum ferritin concentration was distinguished as a potential biomarker for PCOS based on the high area under ROC curve value of 0.71 (95% CI: 0.57–0.86). Although we did not identify a statistical association between serum Zn concentration and PCOS overall, the concentration of Zn in PCOS women with insulin resistance (IR) was lower than that in healthy women (SMD = −0.89, 95% CI: −1.73 to −0.06). Furthermore, the concentrations of Mg (SMD = 0.31, 95% CI: −0.32–0.94, *I*^2^ = 95.4%) and Fe (SMD = −0.59, 95% CI: −1.29–0.12, *I*^2^ = 97.2%) were not statistically significant between the PCOS and control groups. We generated hypothetical pathways for associations among serum Cu, ferritin and PCOS. The serum concentrations of both Cu and ferritin were significantly higher in women with PCOS, and ferritin was identified as a potential early indicator for PCOS screening. Further studies are essential to determine the specific underlying mechanisms.

## Introduction

Polycystic ovary syndrome (PCOS) is a multifactorial and polygenic disorder of the endocrine system characterized by anovulation, hyperandrogenism, and polycystic ovarian morphology (1). According to different diagnostic criteria, the global prevalence of PCOS ranges from 4 to 21% (2) Women with PCOS have significant reproductive effects, including increased risk of infertility, miscarriage, and pregnancy-related complications (3), along with metabolic disorders, such as obesity (4), insulin resistance (IR) (5), and type 2 diabetes mellitus (6). The causes of PCOS are currently unclear and no effective biomarkers for early PCOS screening have been identified to date (7). Recently, alterations in trace element levels in PCOS have attracted considerable research attention (8).

Trace elements, such as zinc (Zn), copper (Cu) and magnesium (Mg), are essential for normal cellular functions, and play major roles in metabolic pathways involving of enzymes, hormones, and vitamins (9). Considerable evidence suggests that abnormal levels of trace elements are associated with metabolic syndrome (10) and PCOS is characteristically accompanied by metabolic dysfunction. However, epidemiological findings on the associations between trace elements and PCOS are inconsistent. For example, Revathi et al. (11) showed that serum levels of Cu and Zn were higher while Mg levels were lower in PCOS patients than the control group. In contrast, Li et al. (12) reported no significant differences in the levels of serum Zn, Mg, and iron (Fe) between PCOS and healthy control groups in a Chinese cohort. A meta-analysis conducted by Spritzer and co-workers in 2015 did not offer a robust conclusion, since only four related articles were included that used the same unit of measurement for specific trace elements (8). In view of the increased related epidemiological evidence in recent years (11, 13, 14), meta analysis data need to be urgently updated. Standardized mean difference (SMD) is a practical meta-analysis statistical method to overcome the inconsistencies in measurement units among different studies (15).

Here, we conducted a meta-analysis of existing publications until 2019 to establish accurate and reliable associations of serum levels of Zn, Fe, Mg, Cu, and ferritin with PCOS and further evaluated the utility of these trace element levels for PCOS screening.

## Materials and Methods

### Search Strategy

We electronically searched PubMed, Embase, and Web of Science using a logical combination of key words. The search terms used were (“polycystic ovary syndrome” OR “poly cystic ovarian syndrome” OR “polycystic ovary disease” OR “polycystic ovaries” OR “polycystic” OR “pcos” OR “stein Leventhal syndrome” OR “SLS”) and (“zinc” or “copper” or “magnesium” or “iron” or “microelement” or “macro elements” or “trace elements”). All articles identified between inception and 26th October 2019 were screened. We also screened the reference lists of these publications for additional references. Conference abstracts were carefully read and screened for unpublished “insignificant results.” We additionally attempted to contact the corresponding authors to request the full text or original data.

### Inclusion and Exclusion Criteria

Studies were included in this meta-analysis if they were confirmed to meet the following inclusion criteria. First, reports needed to be observational studies that included PCOS patients and non-PCOS controls. Moreover, only confirmed PCOS diagnoses were acceptable. Second, studies needed to contain specific data relating to the serum concentrations of Zn, Fe, ferritin, Cu, or Mg, Finally, studies needed to involve humans. Publications were excluded if they were: (1) commentaries, reviews, or conference abstracts, (2) repetitive studies, (3) clinical interventions, (4) animal studies, (5) lacking a control group, and (6) not in English. Additionally, publications reporting data on plasma levels but not serum levels of microelements were excluded.

Based on these factors, the identified titles and abstracts were first independently reviewed by YJ and HX; only relevant publications were selected for full screening and analysis.

### Data Extraction

Two independent reviewers (YJ and JM) extracted a range of data, including the date of publication, first author, study population, study design, sample size, the characteristics of PCOS patients, and the methods used to measure microelements in the serum. We recorded the different concentration units (ng/mL, μg/mL, μg/dL, mg/dL, μg/L, and mmol/L) and the methods used to describe data [mean ± standard deviation (SD), median & interquartile range, median, and range]. All data were rechecked by LR.

### Quality Assessment

We assessed the quality of the included studies in accordance with the Newcastle-Ottawa Scale (NOS) (16). Two reviewers independently scored the NOS grade from three aspects: selection, comparability, and exposure. Any discrepancies between the two reviewers were resolved by reaching a consensus, or by involving a third reviewer (BY).

### Statistical Analysis

All data relating to the concentration of specific trace elements are represented by mean ± standard deviation (SD). We used sample size, median, and range or interquartile range, to estimate the mean and SD using a method that was described previously (17). Since the existing literature is not consistent with regard to units, we used the pooled standardized mean difference (SMD) to determine the associations between PCOS and serum concentrations of microelements. SMD was determined as mean difference divided by standard deviation derived from both groups estimated using Cohen's method (15). The “meta” package in R software was used to estimate effect sizes. Heterogeneity in the studies was tested using Cochran's *Q* two-sided test of homogeneity (18). The *I*^2^ statistic was a crucial factor when determining the model that should be used to pool the effect size (if *I*^2^ < 50%, we used a fixed model, otherwise, we used a random model). Begg's Funnel plots (in cases where the number of included studies was >9) and Egger's regression test were used to test publication bias. The overall strength of evidence was assessed using GRADE criteria (https://gradepro.org/). Sensitivity analyses were performed to test the robustness of the pooled SMD by excluding the study with the largest effect size. Subgroup analyses were conducted according to different diagnostic criteria and sub-classifications of PCOS (e.g., PCOS with obesity, or PCOS with insulin resistance). The receiver operating characteristic (ROC) curve was employed to evaluate the screening value of specific trace elements for PCOS. All analyses were performed using R software. A two-sided *P* ≤ 0.05 was considered to be statistically significant.

## Results

### Study Selection and Characteristics

**Figure 1:**
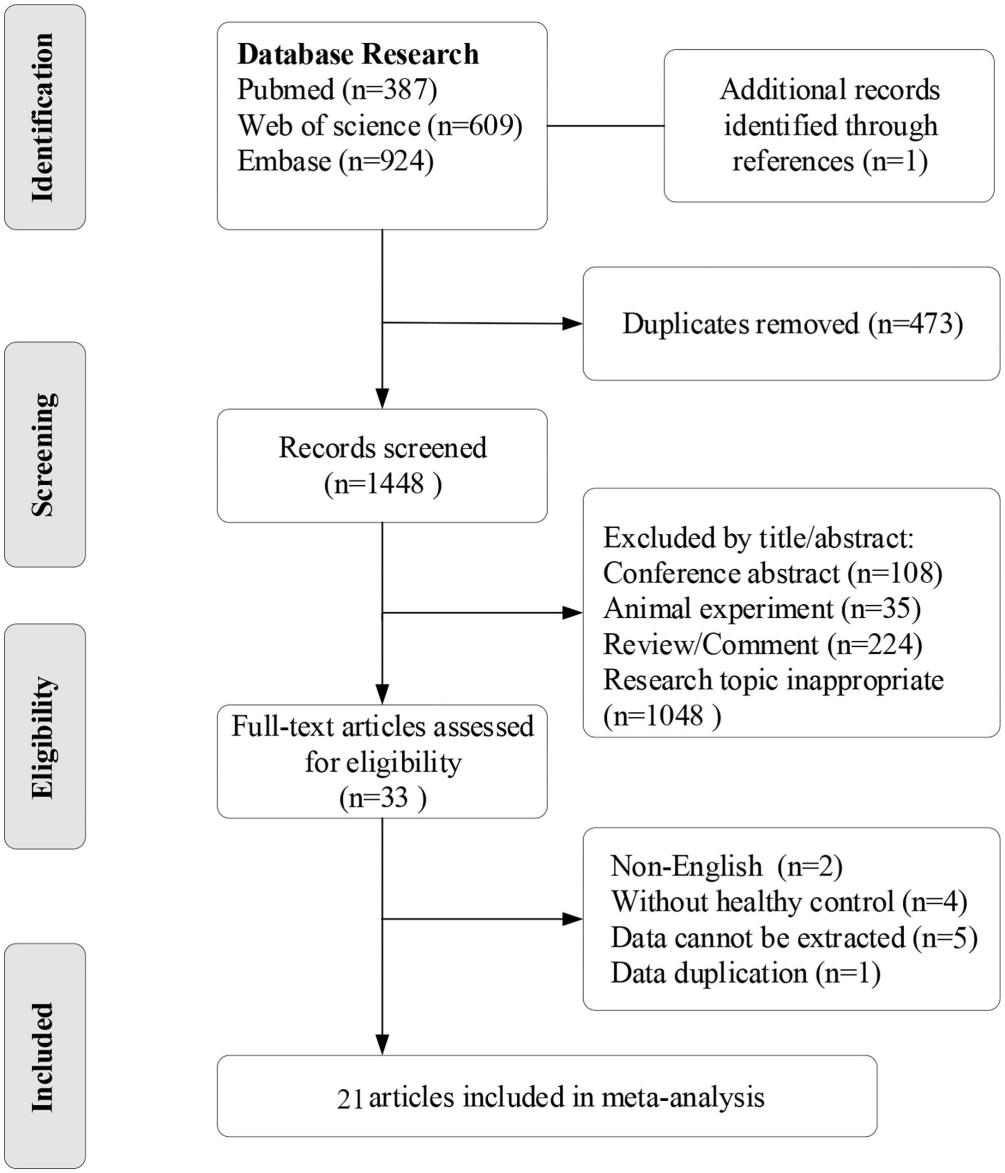
The flow chart of study search and selection.

Our database screening identified 1,921 articles. By removing duplications, and by screening abstracts, we were able to select 33 articles for full-text assessment. In addition, one additional record was identified in the reference list of one of the 33 articles. According to the inclusion and exclusion criteria, there were 21 publications included in our final meta-analysis (11–14, 19–35) (Figure 1). All of these studies featured a cross-sectional design and included individual data from 2,173 women with PCOS and 1,897 healthy controls. The baseline characteristics, such as author, year, country, microelement, units, study design, number of PCOS/control subjects, and specific criteria for PCOS diagnosis included in the studies are shown in Table 1. Three articles separately reported data relating to obese or non-obese PCOS women, (19, 24, 28) while two articles separately reported data relating to IR or NIR PCOS women (14, 30). The overall quality of these articles was relatively high (NOS score ≥6). The specific details are shown in Supplementary Table 1.

**Table 1:** Characteristic of the included studies.

**No**.	**First author**	**Year**	**Country**	**Microelement**	**Unit**	**PCOS/control women(n)**	**PCOS Type**	Diagnostic criteria^*^
1	Luque	2010	Spain	Ferritin	ng/mL	112/86	Lean/Overweight/Obese	A. National institutes of health definition (1990)
2	Escobar	2011	Spain	Ferritin	ng/mL	104/100		A. National institutes of health definition (1990)
3	Luque	2011	Spain	Ferritin	ng/mL	34/30		Clinical and/or biochemical hyperandrogenism, oligoovulation
4	Kauffman	2011	USA	Mg	mmol/L	100/20		B. Rotterdam criteria (2003)
5	Kurdoglu	2012	Turkey	Zn/Mg/Cu	μg/mL	35/30		B. Rotterdam criteria (2003)
6	Sharifi	2012	Iran	Mg	mmol/L	103/103	Normal/overweight/obese	B. Rotterdam criteria (2003)
7	Chakraborty	2013	India	Mg/Cu/Zn	ppm	132/46		B. Rotterdam criteria (2003)
8	Celik	2013	Turkey	Cu	ugr/dL	44/42		C. Modifications based on rotterdam criteria
9	Palomba	2014	Italy	Fe/Ferritin	μg/dL	150/150		B. Rotterdam criteria (2003)
10	Guler	2014	Turkey	Zn	μg/dL	53/33		B. Rotterdam criteria (2003)
11	Yang	2015	China	Ferritin	ng/mL	156/30	Non-obese/obese	B. Rotterdam criteria (2003)
12	Zheng	2015	China	Zn/Cu	μg/L	96/105		B. Rotterdam criteria (2003)
13	Ozer	2016	Turkey	Zn/Cu	μg/dL	71/53	IR/NIR	B. Rotterdam criteria (2003)
14	Li	2017	China	Cu/Zn/Mg/Fe	μmol/L	578/559		B. Rotterdam criteria (2003)
15	Sharif	2017	Sudan	Zn/Cu	μg/mL	50/50		D. Modifications based on rotterdam criteria
16	Hussien	2017	Iraq	Fe/Cu	mg/dL	20/50		Clinical diagnosis history
17	Rashidi	2017	Iran	Fe/Ferritin	μg/dL, ng/mL	56/41		B. Rotterdam criteria (2003)
18	Kanafchian	2018	Iran	Mg/Cu	mg/dL	60/90	IR/NIR	B. Rotterdam criteria (2003)
19	Revathi	2018	India	Cu/Zn/Mg	μg/dL, mg/dL	99/99		B. Rotterdam criteria (2003)
20	Kanafchian	2018	Iran	Zn	μg/dL	60/90		B. Rotterdam criteria (2003)
21	Shahrokhi	2019	Iran	Zn	mg/dL	60/90		Clinical diagnosis history

*Mg, magnesium; Cu, copper; Zn, zine; Fe; IR, insulin resistance; NIR, non-insulin resistance*.

* *A. National Institutes of Health definition (1990): with clinical and/or biochemical hyperandrogenism in addition to oligo-ovulation after excluding secondary etiologies*.

*B. Rotterdam criteria (2003): Meet at least two of the following criteria: (1) hirsutism or hyperandrogenemia in the absence of alternative explanations, (2) oligomenorrhea (≤ 8 cycles per year) or dysfunctional uterine bleeding, (3) polycystic ovaries on ultrasound (12 or more follicles < 10 mm on each ovary or the ovarian volime exceeded 10 cm^3^)*.

*C. Modifications based on Rotterdam criteria: Oligoanovulation was defined as the presence of oligomenorrhea (menstrual cycles of >35 d) or amenorrhea (lack of the menstrual period for 6 month or more)*.

*D. Modifications based on Rotterdam criteria: Oligomenorrhoea was defined as delayed menses>35 days*.

### The Association Between Serum Zinc Concentration and PCOS

**Figure 2:**
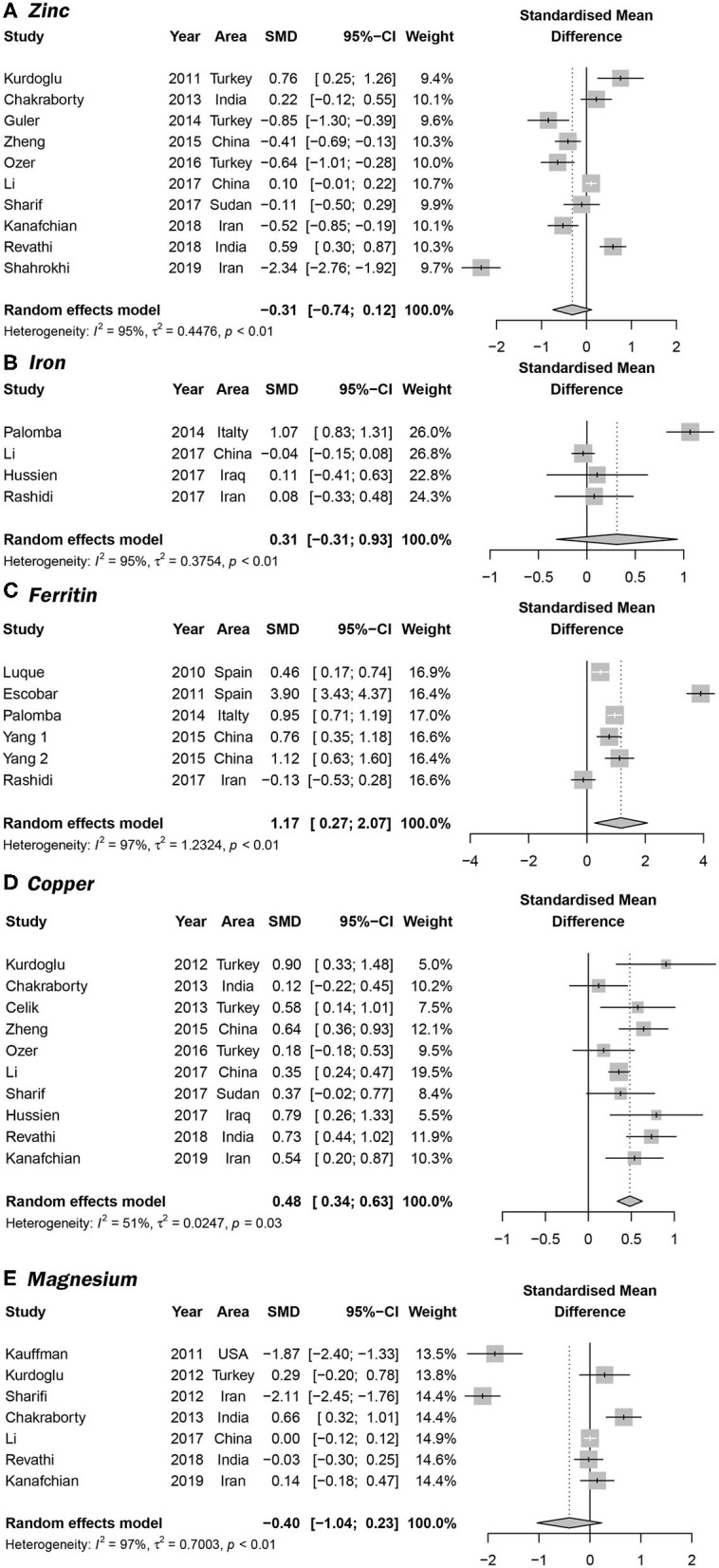
Forrest plots showing serum trace element concentrations in women with PCOS and healthy controls. **(A–E)** represent the association between PCOS and the serum concentrations of serum Zn, Fe, ferritin, Cu, and Fe, respectively.

**Figure 3:**
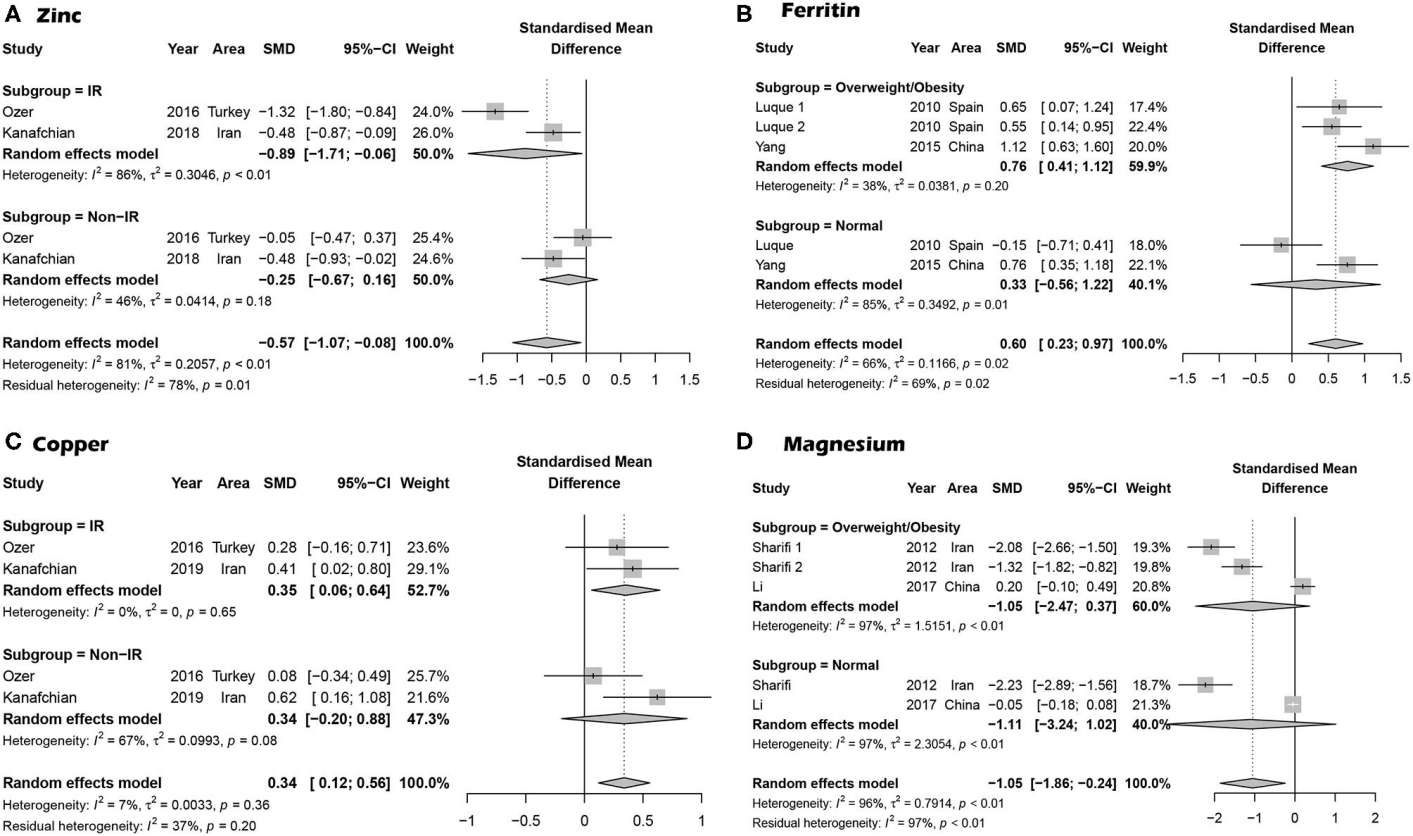
Subgroup analysis based on PCOS with trace elements. **(A)** Subgroup analysis for the association between serum Zn concentration and PCOS based on IR and PCOS. **(B)** Subgroup analysis for the association between serum ferritin concentration and PCOS based on overweight/obese PCOS cases. **(C)** Subgroup analysis for PCOS cases with insulin resistance. **(D)** Subgroup analysis for PCOS patients who were overweight/obese.

Overall, 10 articles focused on the association between zinc concentration and PCOS (11–13, 23, 26, 29, 30, 33–35). We did not find a statistical association between serum zinc concentration and PCOS. The SMD between PCOS and healthy controls ranged from −2.34 (95% CI: −2.76 to −1.92) to 0.76 (95% CI 0.25–1.26) (Figure 2A). Using the random-effects model, pooled SMD was −0.31 (95% CI −0.74–0.12), and *I*^2^ = 95%. Although the Funnel plot presented obvious asymmetry (see Supplementary Figure 2A), publication bias was not statistically significant (Begger test: *P* = 0.175, Egger test: *P* = 0.211). After excluding the results of Shahrokhi et al. (13) (SMD = −2.34), which reported the maximum effect, the pooled SMD remained statistically insignificant (SMD = −0.10, 95% CI: −0.39–0.20) (Supplementary Figure 2B). We further excluded the results of Shahrokhi and Sharif, since they did not use the Rotterdam criteria to diagnose PCOS. The pooled SMD remained insignificant (SMD = −0.09, 95% CI: −0.42–0.24, *I*^2^ = 90%). Subgroup analysis showed that the serum Zn concentration in PCOS women with IR was significantly lower than that of healthy women (SMD = −0.89, 95% CI: −1.71 to −0.06). However, there was no significant difference in serum Zn concentration when compared between healthy controls and PCOS women without IR (SMD = −0.25, 95% CI: −0.67–0.16) (see Figure 3A).

### The Association Between Serum Iron and Ferritin Concentration and PCOS

**Figure 4:**
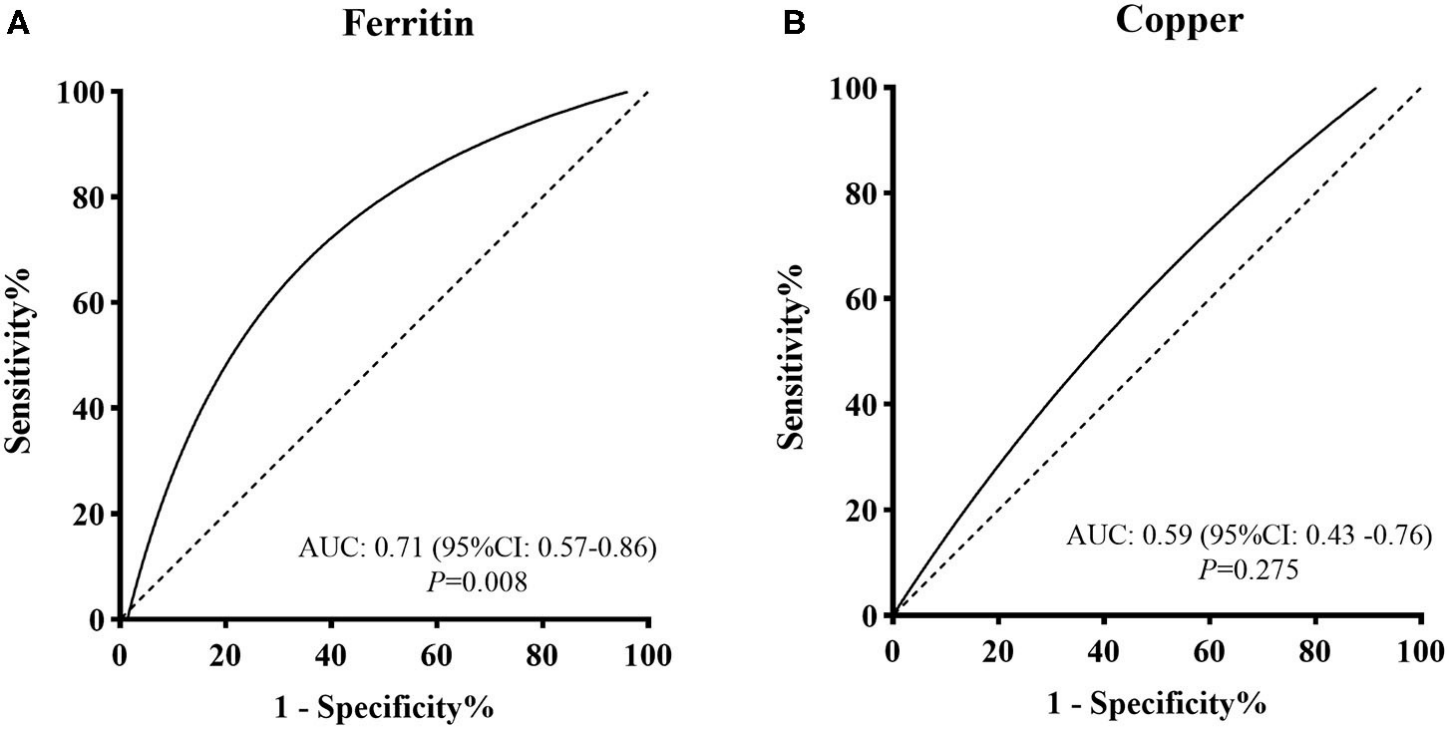
ROC curve evaluations for screening value of **(A)** serum ferritin concentration and **(B)** serum Cu concentration for PCOS.

Four studies focused on serum Fe concentrations in patients with PCOS (12, 27, 31, 32), while 5 studies focused on ferritin concentrations (19, 20, 27, 28, 32). The serum concentration of Fe in PCOS patients was not significantly different from that in healthy controls (SMD = 0.31, 95% CI: −0.31–0.93, *I*^2^ = 95%) (Figure 2B). There was no significant publication bias with regards to these publications (Begger test: *P* = 0.734; Egger test: *P* = 0.601). The SMD of serum ferritin concentration between PCOS and healthy controls ranged from −0.13 (95% CI: −0.53–0.28) to 3.90 (95% CI: 3.43–4.37) (Figure 2C). The pooled SMD was 1.17 (95% CI: 0.27–2.07, *I*^2^ = 97.0%), indicating that the serum concentration of ferritin in PCOS patients was higher than that of healthy women. This association was confirmed with moderate evidence (Supplementary Table 6). Begg's (*P* = 0.734) and Egger tests (*P* = 0.601) revealed no significant publication bias. After excluding the results of Escobar et al. (20), pooled SMD was also < 0 (SMD = 0.63, 95% CI: 0.24–1.03) (Supplementary Figure 3). We further excluded the results of Luque and Escobar, since they did not use the Rotterdam criteria to diagnose PCOS. The pooled SMD remained significant (SMD = 0.68, 95% CI: 0.16–1.19, *I*^2^ = 87%). Subgroup analysis further showed that among overweight/obese women with PCOS, the serum concentration of ferritin was greater than that of healthy women (SMD = 0.76, 95% CI: 0.41–1.12); there was no such increase in serum concentrations of ferritin in women with PCOS who were within the normal weight range (SMD = 0.33, 95% CI: −0.56–1.22) (Figure 3B). Data obtained using the ROC curve suggest that the serum ferritin concentration could be effectively used to distinguish between PCOS and healthy controls to some extent (Figure 4A; area under the curve (AUC) = 0.71, 95% CI: 0.57–0.86).

### The Association Between Serum Copper Concentration and PCOS

We identified 10 articles that focused on the serum concentration of Cu in women with PCOS (11, 12, 14, 23, 25, 29–31, 33, 35). The SMD for serum Cu concentration between PCOS and healthy controls ranged from 0.12 (95% CI: −0.22–0.45) to 0.90 (95% CI: 0.33–1.48) (Figure 2D). Using a random effects model, the pooled SMD was 0.48 (95% CI: 0.34–0.63) and *I*^2^ = 51%; this indicated that the serum concentration of Cu in PCOS patients was higher than that of healthy women. This association was verified by moderate evidence (Supplementary Table 6). We observed no significant publication bias (Supplementary Figure 4, Begger test: *P* = 0.348, Egger test: *P* = 0.080). We further excluded the results of Celik, Sharif and Hussien since they did not use the Rotterdam criteria to diagnose PCOS. The pooled SMD remained significant (SMD = 0.47, 95% CI: 0.29–0.64, *I*^2^ = 63%). Subgroup analysis showed that among PCOS women with IR, the serum concentration of Cu was greater than that of healthy controls (SMD = 0.35, 95% CI: 0.06–0.64); the SMD was not significant when we analyzed PCOS patients without IR (SMD = 0.34, 95% CI: −0.02–0.88) (see Figure 3C). Nevertheless, data from ROC curve analysis indicated that the predictive value of serum Cu was not statistically significant (Figure 4B; AUC = 0.59, 95% CI: 0.43–0.76; *P* = 0.275).

### The Association Between Serum Magnesium Concentration and PCOS

We identified 7 articles that focused on the serum concentration of Mg in women with PCOS (11, 12, 14, 21, 23, 24, 35). All the included studies used the Rotterdam criteria for PCOS diagnosis. No significant differences in the serum concentration of Mg were evident between PCOS patients and healthy controls (SMD = −0.40, 95% CI: −1.04–0.23). Further analysis revealed high levels of heterogeneity among the 7 articles (*I*^2^ = 97%); the SMD ranged from −2.11 (95% CI: −2.45 to −1.76) to 0.66 (95% CI: 0.32–1.01) (Figure 2E). Begg's test (*P* = 0.260) and Egger test (*P* = 0.320) showed no significant publication bias. After excluding the studies of Sharifi (33) and Kauffman (21), which reported relatively extreme results, the pooled SMD was −0.07 (95% CI: −0.02–0.17) (Supplementary Figure 5). Subgroup analysis further showed that the serum concentration of serum Mg was not significantly different between PCOS patients and healthy controls, irrespective of whether or not PCOS patients were obese (overweight/obese: SMD = −1.05, 95% CI: −2.47–0.37; normal weight: SMD = −1.11, 95% CI: −3.24–1.02) (Figure 3D).

## Discussion

Knowledge of the potential associations of trace elements with PCOS occurrence and development should provide effective new strategies to prevent, screen and treat PCOS, which has public health significance. Here, we identified 21 specific articles on the associations between PCOS and serum concentrations of Zn, Mg, Cu, Fe, and ferritin. The results showed that PCOS patients had significantly higher serum concentrations of Cu and ferritin than healthy controls. However, no significant differences were observed with regard to the levels of Zn, Fe, and Mg between the PCOS and control groups. Our report provides not only an update on meta-analysis data but also preliminary evidence of the screening value of serum ferritin concentration for PCOS.

Our results showed that serum Cu and ferritin are associated with PCOS. Cu is an essential trace element in the human body and required as a cofactor for a range of enzymes in critical metabolic pathways, including cytochrome oxidase, superoxide dismutase, ascorbic acid oxidase, and tyrosinase (36). Recent studies have shown that Cu interacts with key neuropeptides in the hypothalamic-pituitary-gonadal axis, notably, gonadotropin-releasing hormone (GnRH) and neurokinin B, and promotes anovulatory menstruation (37). Excessive levels of Cu induce oxidative stress via Fenton and redox reactions, resulting in increased production of reactive oxygen species (ROS) (38). A previous study showed significantly higher levels of oxidative stress parameters, including total antioxidant and oxidant status and oxidative stress index, in PCOS patients than healthy controls (39), indicating a role of oxidation in the pathogenesis of the disease. ROS can alter the steroidogenesis process in the ovary, leading to increased androgen levels, disturbance in follicular development, and infertility (40). Moreover, IR is reported to be linked with oxidative stress, which may mediate PCOS occurrence through facilitating secretion of excessive levels of androgens from ovaries and adrenal glands (41).

**Figure 5:**
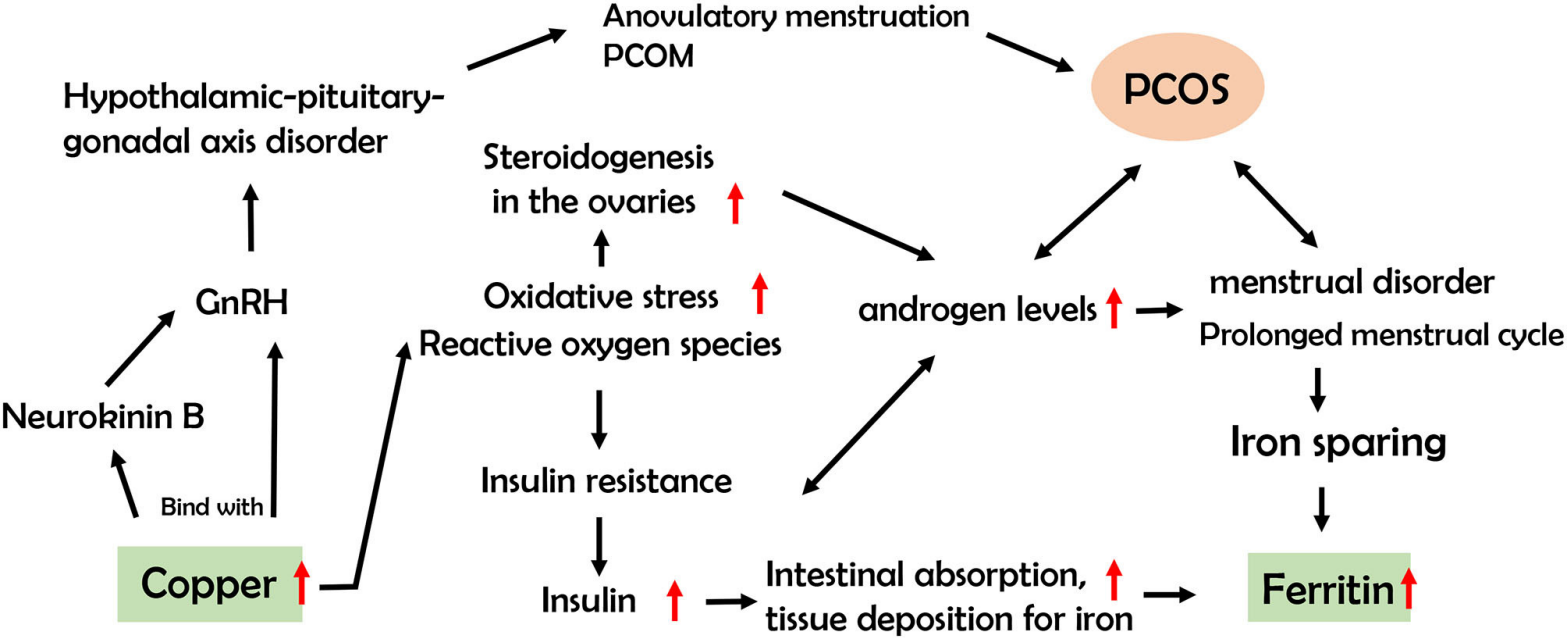
Hypothetical pathways for the associations among serum Cu, ferritin, and PCOS.

Ferritin, the cellular storage protein for iron, serves as a biomarker for estimating the levels of iron stored in the body. Several factors potentially contribute to elevation of serum ferritin levels in women with PCOS, including the iron-sparing effect caused by prolonged menstrual cycle and hyperinsulinism (42). Meanwhile, higher insulin may facilitate intestinal absorption and deposition of iron in tissue, with IR leading to higher levels of ferritin (42). Our results also showed an association of obese/overweight PCOS subjects with higher serum ferritin but not those with normal BMI, indicating a critical role of overweight/obesity. This finding was consistent with that of Hitha et al. (43), which showed a significant positive relationship between ferritin and metabolic parameters in obese subjects. Although serum Fe showed a similar increasing trend among PCOS subjects, the data were not statistically significant, suggesting that the serum Fe level may be a less sensitive parameter than ferritin. Based on the collective findings, a hypothetical pathway was drawn to describe the potential associations among Cu, ferritin and PCOS (Figure 5).

The complex effects of trace elements on body functions may partly explain the inconsistency of epidemiological results. Zn acts as a stabilizer and cofactor for many enzymes and is an essential element for hormonal function (44). In addition, Zn is a regulator of islet function and glucose homeostasis and combines with insulin hexamers to promote the stability and binding ability of insulin receptors (45, 46). Although no association with PCOS has been established, Zn supplementation is reported to ameliorate insulin sensitivity, improve glucose homeostasis, and alleviate insulin resistance (47–49). Mg is involved in over 300 enzyme systems and has been identified as a necessary nutrient for energy production and synthesis of nucleic acids. Considerable evidence suggests that IR can be improved in women with PCOS following Mg supplementation (50, 51), but the specific mechanisms are still unclear.

To explore the causal correlations between trace elements and PCOS, many randomized controlled trials (RCTs) have been performed to establish whether trace element supplements have beneficial effects on PCOS treatment (52, 53). The group of Afshar (53) showed that Mg and Zn co-supplementation decreased serum high-sensitivity C-reactive protein and increased plasma total antioxidant capacity levels. However, inconsistent results have been obtained from different studies (52). Notably, increases in Cu and ferritin were difficult to adjust through simple supplements. To our knowledge, no RCTs have focused on the significance these elements in PCOS. Identification of practical biomarkers to screen for PCOS among childbearing women remains an urgent medical requirement. PCOS is a clinical outcome of long-term changes in the endocrine system (54). We assume that subtle alterations do not raise clinical concerns, including changes in trace elements, which may slowly cause PCOS. Here, we reported the screening value of serum ferritin for PCOS for the first time, which requires further verification. Our findings were similar to the results of Spritzer et al. (8). Our study provides more robust evidence since a larger number of studies were included, some of which were published in recent years. Additionally, we addressed two problems reported by the group of Spritzer. SMD was employed to overcome the challenge of heterogeneity of measurement units used among different studies (15). The method of Wan (17) was used to process non-normal data. Despite the possibility of introducing greater heterogeneity, comparison of data from different sources could provide valuable information.

A number of limitations in our study should be acknowledged. First, heterogeneity existed among the original articles due to differences in participant backgrounds and methods used to detect trace elements. Second, SMD was used to estimate the difference, which simply reflected the variation trends of trace elements among PCOS but not the actual levels, that would impact clinical application. Third, the cross-sectional or case-control designs of original articles would limit causal inference. We could not conclude whether the changes in trace elements induce PCOS or exert a converse effect. Fourth, the sample sizes of studies focusing on trace element analysis were small, potentially resulting in bias of results. Fifth, due to data limitations, the main confounding variables were not adjusted for and we could not analyze the possible confounding effect of obesity or IR on all associations through sub-group analysis. These factors could have influenced our final comparative analyses. Furthermore, only literature published in English was included. Although a number of researchers propose that the language of publication has little effect on the pooled effect estimates (55), the possibility of publication bias cannot be overlooked. Potential unpublished data may additionally contribute to publication bias.

In conclusion, serum concentrations of Cu and ferritin are significantly higher in subjects with PCOS. Moreover, ferritin may serve as an early indicator of PCOS screening. Further studies are required to investigate the significance of other elements, including Mg, Zn and Fe, in PCOS and the specific mechanisms involved.

## Data Availability Statement

All the original data were presented in the main text and Supplemental Materials. Any other questions can contact the corresponding author: Ran Liu, ranliu@seu.edu.cn.

## Author Contributions

JY, XH, and JM: literature search, screening, and data extraction. XH and JY: data analysis and results visualization. JY, XH, YB, and RL: manuscription draft and modification. RL and YB: fund acquirement. All authors reviewed the final version of the manuscript and approve it for publication.

## Conflict of Interest

The authors declare that the research was conducted in the absence of any commercial or financial relationships that could be construed as a potential conflict of interest.

## Abbreviations

PCOS: Polycystic ovary syndrome
IR: insulin resistance
ROS: reactive oxygen species
Zn: zinc
Cu: copper
Mg: magnesium
Fe: iron
SMD: standard mean difference.
